# Assisted Reproductive Technology: Gaps in young adults’ perceptions and information from clinics: José Miguel Carrasco

**DOI:** 10.1093/eurpub/ckac129.588

**Published:** 2022-10-25

**Authors:** M López-Toribio, J Melovska, V Dimitrievska, A Dostálová, M Novotná, V Rozée, K Hens, S March, F Güell, JM Carrasco

**Affiliations:** 1 Research and Transfer, APLICA Cooperative, Madrid, Spain; 2 Qualitative Research, Healthgrouper, Skopje, Republic of North Macedonia; 3 Management, Medistella Mediversvm s.r.o., Prague, Czechia; 4 Sexual and Reproductive Health and Rights, Institut National d’Etudes Démographiques, Paris, France; 5 Department of Philosophy, University of Antwerp, Antwerp, Belgium; 6 Mind-brain Group, Institute for Culture and Society ICS-UNAV, Pamplona, Spain

## Abstract

**Background:**

Around 186 million individuals are facing infertility worldwide, with a huge impact on their wellbeing. Fertility care is considered a key element to promote reproductive health. This study aims to explore young adults’ knowledge, perceptions and concerns about infertility and Assisted Reproductive Technology (ART), and to contrast it with the information provided by ART clinics.

**Methods:**

A multi-country qualitative study was conducted in Albania, Belgium, Slovenia, Spain, Italy, Kosovo, North Macedonia and Switzerland within the H2020 B2-INF project (Grant Agreement 872706). In 2021, 10-15 semi-structured interviews were conducted in each country with participants aged 18-30, childless and non-ART users. Additionally, 3-5 clinics’ websites in each country were explored. Data was collected in native languages and translated into English. A thematic analysis was carried out.

**Results:**

In total, 98 interviews were conducted and 38 clinics’ websites were explored. Three themes emerged from the analysis of the interviews: 1. Parenthood and (in)fertility; 2. Young people's perception on ART; 3. Information and publicity of ART. Parenthood was described as a relationship beyond biological ties and infertility as a social taboo. Most participants perceived ART positively and would use it if needed, although knowledge on ART was low. Participants considered information on ART as scarce and suggested that government-led information campaigns should be launched to raise awareness on it. Concerning clinics, the websites provided information on infertility and exhaustive technical descriptions of ART techniques, although it may be difficult for the general population to understand. Data offered on success rates were unclear and heterogeneous.

**Conclusions:**

Gaps in citizens’ expectations and needs and the information provided by ART clinics were identified. Awareness campaigns are needed to diminish social taboo on infertility and ART and to promote reproductive health.

**Key messages:**

• Assisted Reproductive Technology is positively perceived by young adults, though their knowledge of it and infertility is low. Information campaigns are thus needed to raise awareness among the young.

• ART clinics have room for improvement in aligning the information provided on their websites with young people's perceptions and needs, and to show it in a more accessible and understandable way.

